# The Risk and Benefits of Various Innovations in Fusion and Fusionless Surgery for AIS

**DOI:** 10.3390/jcm12134552

**Published:** 2023-07-07

**Authors:** Tom P. C. Schlösser, Per D. Trobisch

**Affiliations:** 1 Department of Orthopaedic Surgery, University Medical Center Utrecht, Postbus 85500, 3508 GA Utrecht, The Netherlands; t.p.c.schlosser@umcutrecht.nl; 2 Department of Spine Surgery, Eifelklinik St. Brigida, Kammerbruchstr. 8, 52152 Simmerath, Germany

Adolescent idiopathic scoliosis is the most common variety of the condition, constituting a three-dimensional deformity of the spine and chest that primarily affects otherwise healthy adolescents. If severe progressive curves remain untreated, scoliosis patients have a poor prognosis that comes with a substantial life-long burden of disease due to the deformation of the spine and chest, restrictions in the area of the pulmonary system, and sometimes debilitating pain.

The treatment of AIS attempts to alter the natural course of the disease. Since the 1980s, posterior spinal fusion (PSF) has been widely adapted as the standard treatment for AIS patients at the end of adolescent growth with severe progressive curves owing to its capacity to prevent further deformation and to partially correct spinal and chest deformity. The primary goal of PSF for scoliosis has always been to prevent progression and to provide a balanced spine in the coronal and sagittal plane, fuse as few vertebrae as possible and avoid complications. Correcting a scoliotic spine to possess as normative an anatomy as possible is beneficial to the remaining unfused areas of the spine. Even with contemporary surgical techniques, it remains difficult to really recreate the normal shape of the spine and avoid problems like proximal junctional kyphosis, shoulder imbalance or degenerative disc disease.

Especially in patients with structural proximal thoracic curves (Lenke type 2), shoulder balance remains a challenge in PSF. In a recent retrospective study on the Harms Study Group Multicenter AIS Database, Landrum et al. validated a tool to aid in case-specific determinization of the optimal amount of coronal plane correction for the proximal thoracic as compared to the main thoracic curvature [1]. The thoracic curve correction ratio is a novel parameter that combines the pre-operative mismatch between the curves to the intra-operative correction of both curves, and has significant correlation with T1tilt. While the application of these parameters demonstrates their relevance to treating radiographic shoulder (im)balance, further studies must consider the appearance and functional perspective of patients.

For corrective scoliosis surgery, it is essential to have thorough understanding of the effect of correction of the deformity in all three anatomical planes. In general, successful restoration of coronal and sagittal balance has a protective effect on the unfused discs in posterior spinal fusion for AIS. In the sagittal plane, patients with AIS typically have regional thoracic hypokyphosis and compensatory decreased cervical lordosis. If the surgeon performing three-dimensional correction maneuvers focusses on coronal and axial correction, due to the nature of the deformity, the sagittal plane is corrected to a lesser extent. Persistent thoracic hypokyphosis may drive the cervical spine into further kyphotic compensation, with potentially negative effects on the unfused cervical discs. Interestingly, Young et al. surveyed 180 AIS patients treated between 1975 and 1992 by Milwaukee braces comprising Harrington rods, and 33 had lateral radiographs available at adolescent age [2]. At an average follow-up age of 43 years, 4 AIS patients had undergone cervical surgery for disc disease (2.2%), compared to 0.03–0.16% of the general population. This unique historical cohort supports the idea that the association between sagittal alignment after previous treatment for AIS, long-term cervical degeneration, and patient-related outcomes should be further explored.

PSF does not cure scoliosis, the physiological motion of the instrumented part of the spine is not preserved and patients have reduced functional outcomes as compared with the normal population. While PSF for AIS provides sustainable long-term outcomes, the surgical field remains highly innovative. The disadvantages of spinal fusion have led surgeons to study other surgical methods for correcting AIS without fusion. To control the asymmetric growth of the vertebral bodies and intervertebral discs, vertebral body tethering (VBT) has been introduced as motion preserving alternative to address progressive scoliotic deformities. Using PRISMA guidelines for systematic reviews, Raitio et al. synthesize the published results about this technique and discuss the indications, timing, outcomes and complications of VBT [3]. They conclude that VBT allows for the correction of scoliotic deformity and preserves motion in moderately sized curves in children before the peak of the adolescent growth spurt. As no randomized trials or prospective follow-up studies are available to compare VBT to PSF, however, the authors also conclude that evidence-based recommendations on VBT are lacking.

As of yet, the complication and reoperation rate of VBT is still high. Surgeons who are planning to start VBT are often aware of the potential pulmonary complications, which are very rare after PSF. In order to shed light on the pulmonary complications involved in VBT, Trobisch et al. described a pulmonary complication rate of 10% in a retrospective single-center cohort study (n = 140), with no long-term consequences [4]. Interestingly, the majority were diagnosed after discharge and between 2–6 weeks postoperatively. 

As VBT aims to correct the scoliotic deformity by growth modulation of the spinal tissues, the use of VBT after the peak of the growth spurt is controversial. Recently, Meyers et al. described the outcomes of VBT for 49 adolescents with a mean age of 15 years, relative skeletal maturity (Risser 3–5), and a minimum of a 2-year follow-up. While 41% of the tethers broke during follow-up period, VBT resulted in satisfactory clinical and radiological outcomes in this population [5]. This raises the question: does VBT require growth modulation to be successful, or can it hold the spine straight long enough to allow for sufficient remodeling of the intervertebral discs and bone such that a long-term spinal correction can be maintained. 

In contrast to adolescent scoliosis, early-onset scoliosis requires growth-friendly treatment to allow the spine and chest to grow to adult proportions. As PSF and VBT do not allow for extensive growth, Tabeling et al. recently presented a novel growth-friendly system for treating early-onset scoliosis [6]. In contrast to traditional growing rods, by the application of one or two springs around rods in sliding connectors, the scoliosis is corrected by continuous distraction forces and the spine is kept dynamic. In their prospective study, the authors present the positive effect of design iterations on lowering implant-related complication rates to levels lower than previous designs and better than magnetically controlled growing rods, while maintaining similar spinal corrections compared to previous designs.

These six innovative studies on optimization of PSF, VBT and surgery for early-onset scoliosis demonstrate that the ultimate goal of scoliosis surgery is evolving rapidly. The provision of balance, the sparing of motion, and the preservation of growth can be added to the list of aims alongside the prevention of further curve progression. The benefits and potential risks of surgical innovations, however, should be monitored closely.
